# Creating a training set for artificial intelligence from initial segmentations of airways

**DOI:** 10.1186/s41747-021-00247-9

**Published:** 2021-11-29

**Authors:** Ivan Dudurych, Antonio Garcia-Uceda, Zaigham Saghir, Harm A. W. M. Tiddens, Rozemarijn Vliegenthart, Marleen de Bruijne

**Affiliations:** 1 Department of Radiology, University of Groningen, University Medical Centre Groningen, Groningen, Netherlands; 2 Department of Radiology and Nuclear Medicine, Erasmus MC, Rotterdam, Netherlands; 3 Department of Paediatric Pulmonology and Allergology, Erasmus MC-Sophia Children Hospital, Rotterdam, Netherlands; 4 Department of Medicine, Section of Pulmonary Medicine, Herlev-Gentofte Hospital, Hellerup, Denmark; 5 Department of Clinical Medicine, University of Copenhagen, Copenhagen, Denmark; 6 Department of Computer Science, University of Copenhagen, Copenhagen, Denmark

**Keywords:** Artificial intelligence, Image processing (computer-assisted), Respiratory system, Thorax, Tomography (x-ray computed)

## Abstract

Airways segmentation is important for research about pulmonary disease but require a large amount of time by trained specialists. We used an openly available software to improve airways segmentations obtained from an artificial intelligence (AI) tool and retrained the tool to get a better performance. Fifteen initial airway segmentations from low-dose chest computed tomography scans were obtained with a 3D-Unet AI tool previously trained on Danish Lung Cancer Screening Trial and Erasmus-MC Sophia datasets. Segmentations were manually corrected in 3D Slicer. The corrected airway segmentations were used to retrain the 3D-Unet. Airway measurements were automatically obtained and included count, airway length and luminal diameter per generation from the segmentations. Correcting segmentations required 2–4 h per scan. Manually corrected segmentations had more branches (*p* < 0.001), longer airways (*p* < 0.001) and smaller luminal diameters (*p* = 0.004) than initial segmentations. Segmentations from retrained 3D-Unets trended towards more branches and longer airways compared to the initial segmentations. The largest changes were seen in airways from 6th generation onwards. Manual correction results in significantly improved segmentations and is potentially a useful and time-efficient method to improve the AI tool performance on a specific hospital or research dataset.

**Supplementary Information:**

The online version contains supplementary material available at 10.1186/s41747-021-00247-9.

## Key points

- Artificial intelligence (AI) segmentation tools require high-quality training data matching the population and scanning parameters of the use case.
- Manually correcting initial airway segmentations based on free tools is an efficient way to create an optimal dataset for AI training purposes.
- Performance of an existing AI model trended towards more complete airways following retraining with corrected data.

## Background

Airway segmentation from computed tomography (CT) scans is important in the study of pulmonary disease such as chronic obstructive pulmonary disease (COPD) [1]. High-quality airway segmentation datasets are difficult to create, yet they are necessary for the training of artificial intelligence (AI) tools. Manually segmenting airways from noisy low-dose CT scans is time consuming and error prone, and methods that can provide adequate large airway segmentation via region growing may fail and require manual correction [2, 3].

The volume of thoracic CT scans in clinical care will increase due to an increasing respiratory disease burden and the introduction of imaging-based cancer screening [4]. Computer assistance will become increasingly important in the radiology workflow. This should be supplemented with robust AI tools that can increase the accuracy and speed of diagnosis. Medical datasets used to train AI tools are typically small, due to the limited availability of imaging data and ground-truth annotations. In contrast, there is a wide range in possible CT scanning and population characteristics. Thus, pre-trained AI tools have issues generalising when tested on new data, with typically different characteristics. In such a setting, the need for quickly adapting an existing AI model trained on different data may prove very useful.

AI segmentation tools are being widely studied for their potential in automation, accuracy, and reliability; however, their use comes at the cost of flexibility inherent in AI systems. To achieve the highest accuracy, AI requires training on scans like those it will be used on. X-ray tube current, voltage, reconstruction methods and other parameters change the resulting CT image and may have an impact on segmentation performance [5].

So far, the methodology for obtaining high quality ground truth segmentations of airways using openly available tools is lacking. Whilst many airway segmentation tools already exist, those that provide a highly detailed segmentation may be only available for sale, are run as a service or tied to specific CT scanner brands and hospital/research setup [6, 7].

In this study, we propose a solution to prepare good ground-truth segmentations by improving the airway segmentations that were obtained using openly available tools and investigate the change in AI performance on our low-dose chest CT protocol following re-training using the corrected segmentations [8].

## Methods

### Initial segmentations

We used a 3D-Unet method [9, 10] designed for automatic airway segmentation. The 3D-Unet is a deep-learning model for biomedical image segmentation, which classifies image voxels as airway/non-airway. The image filters in the convolution layers of the method were optimised automatically using training images and reference segmentations. For all our experiments, we used the same 3D-Unet model layout and hyperparameters as in [9], which were found to be well-suited for airway segmentation.

The current 3D-Unet was trained on Danish Lung Cancer Screening Trial (DLCST) [11] and Erasmus MC-Sophia data (paediatric cystic fibrosis patients) [12]. This model was used to obtain initial airway segmentations from scans of fifteen randomly selected participants from the ImaLife study [8]. The CT scans used were low-dose unenhanced, obtained using a 16-slice CT scanner (Somatom Sensation 16, Siemens Medical Solutions) with a pitch of 3 (with FOV 350) or 2.5 (with FOV 400) and 1 mm increments at a tube voltage of 120 kVp and reference current of 20 mAs [13].. Images were reconstructed with overlapping 0.7-mm increments using the Qr59 kernel. The ImaLife study is part of the northern Netherlands’ study and includes participants of at least 45 years of age from the general population. Complete details on ImaLife patient characteristics can be found in Table S1 and the referenced material [8]. Differences in population and scanning parameters for DLCST and ErasmusMC datasets compared to ImaLife dataset contributed to incomplete initial segmentations. The prediction threshold of the 3D-Unet probability maps was set to 0.5, which resulted in a low number of false positive airways in the initial segmentations so that most corrections required addition of missing branches, rather than removal of false branches.

### Manual correction of segmentations

Initial segmentations were imported into 3D Slicer 4.1 (http://www.slicer.org) [14]. Window settings were set to a width of 800 and a level of − 625 to better visualise the airway lumen. One medical doctor with 6 months of work and training in pulmonology (I. D.) performed the manual corrections of segmentations.

**Fig. 1:**
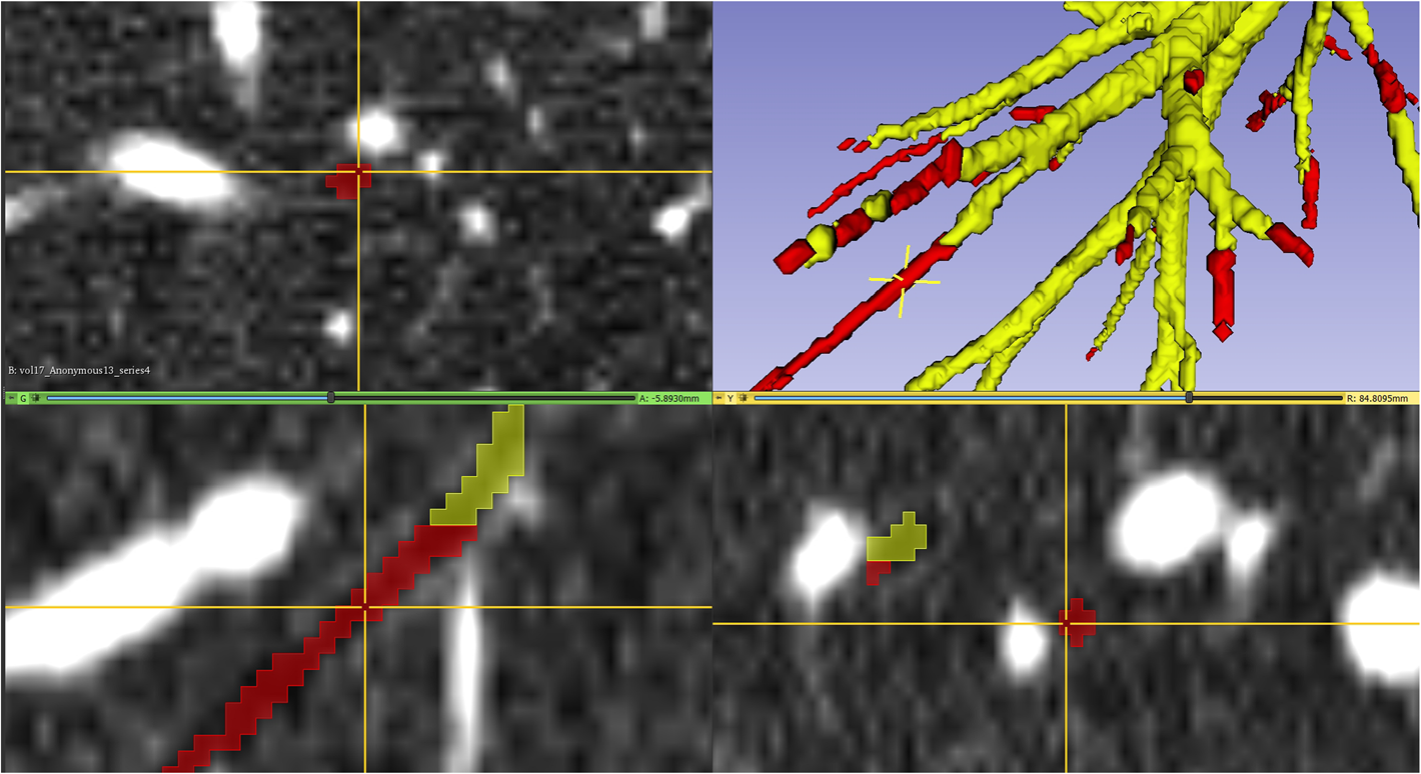
A 3D Slicer workspace for fast identification and correction of incomplete airways. Yellow: incomplete airway segmentation of an ImaLife participant. Red: manual correction of the airway

The workflow screen displayed the coronal, sagittal and transverse and three-dimensional (3D) views (Fig. 1). Corrections were performed using the segment editor tool in 3D Slicer [14]. The binary segmentation provided by the 3D-Unet was imported into the segment editor. Next, the airways segmentations were completed using the paint tool, with a spherical brush and brush size dynamically set to 1–3% of the active window size, based on the size of the airway. 3D Slicer provides tools to follow along an incomplete airway in the 3D view and identify it on the three views. In this manner, it was possible to quickly complete airway segmentations as they were identified on all three orientations simultaneously, with the results instantly visible on the 3D view.

The initial segmentation was combined with the corrections and exported as a set of DICOM slices. A standard operating procedure is provided in the supplemental materials, explaining the process in detail (Electronic Supplementary Material Manual).

### 3D-Unet evaluation

We used the 15 corrected ImaLife scan segmentations to train a new 3D-Unet, referred to as ‘retrained’ model. For training and evaluation, we used a 5-fold cross-validation setting, splitting the dataset into 5 groups of equal size, and training 5 different models, assigning for each model one split group as testing set, and using the remaining 4 of the 5 data as training set. Within each training fold, 83% of data is used for model weight updating, and the remaining 17% for model selection. We evaluate each trained model on their corresponding independent testing set. Each training fold contains 12 scans. Despite the small number, the 3D-Unet [9] was validated with varying sizes of training sets and the learning curves show good performance with similar numbers of scans.

To assess the AI performance by introducing a larger set of heterogeneous data, we trained a second model with a combination of ImaLife, DLCST and ErasmusMC data, referred to as ‘combined’ model. We used the same 5-fold cross-validation split of the ImaLife data as for the ‘retrained’ model above, adding 20 scans each from DLCST and ErasmusMC to the training folds. Trained models were used to segment airways from ImaLife scans for comparison to the initial segmentations. The overall process is summarised in the flowchart shown in Fig. S1.

### Analysis of segmentations and statistical analysis

From the segmentations obtained by the 3D-Unet, branches and their generation number were extracted automatically, similarly to methods used in the EXACT ’09 paper [2]. The airway generation was defined as the number of branch bifurcations counted in the path linking the given branch and the first branch in the airway tree, i.e., the trachea. Thus, the trachea is generation 0, main bronchi generation 1, etc. Automatic measurements of lumen diameter were obtained every 1 mm along the centreline of and averaged per branch. The branch length was calculated as the distance between bifurcations along the centreline of a branch.

Comparison was made between the initial segmentations and segmentations from the retrained and combined models trained with the manually corrected segmentations. Results were analysed using Python (Python Software Foundation, https://www.python.org/) and the SciPy package [15]. Wilcoxon signed rank test with Bonferroni correction was used for analysis. All comparisons were to the initial, incomplete segmentations. A *p* value lower than 0.05 was considered significant.

## Results

### Segmentations

**Fig. 2:**
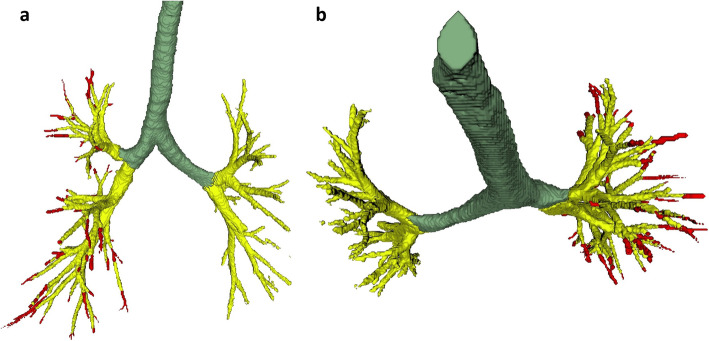
An example of an incomplete segmentation of an ImaLife participant’s airway tree (in yellow) of the left lung and a manually corrected segmentation (in red) of the right lung

**Fig. 3:**
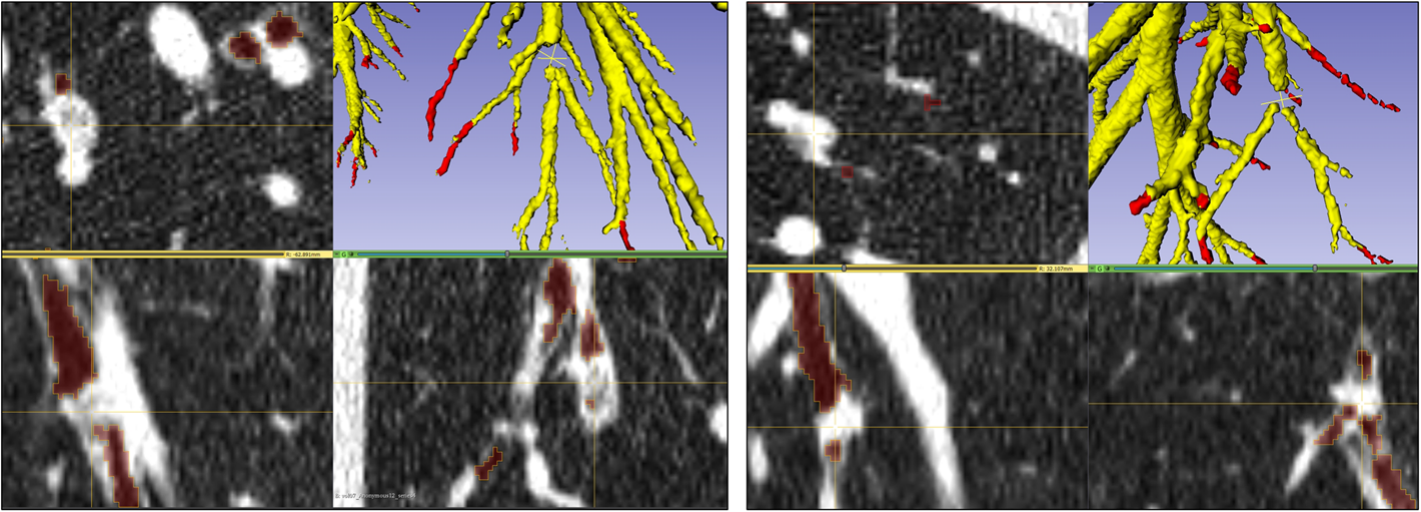
Two examples of large mucous plugging with total focal occlusion of the airway of an ImaLife participant. The 3D-Unet completed segmentation of branches distal to the occlusion without supervision

Fifteen ImaLife scans were segmented by the initial 3DUnet and manually corrected (Fig. 2). In two cases of large mucous plugging, the 3D-Unet continued to segment the airways beyond the blockage without the need for manual interaction (Fig. 3). The time to complete a manual correction ranged from 2 to 4 h.

### Airway count

**Fig. 4:**
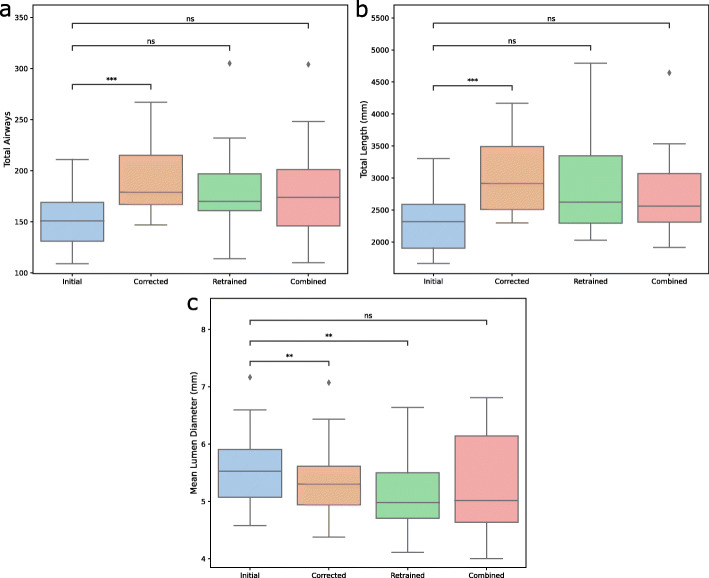
Boxplots for retrained and for combined retrained 3D-Unets. **a** Total airway count per segmentation. **b** Total airway length per segmentation. **c** Median luminal diameter per segmentation. *ns* not significant, *p < 0.05, **p < 0.01, ***p < 0.001

The initial, incomplete segmentations had the lowest median count of 151 airways (interquartile range [IQR] 131–169) followed by the retrained model segmentation with 170 airways (IQR 161–197) (*p* = 0.098, initial vs retrained), the combined model segmentation with 174 airways (IQR 146-201) (*p* = 0.089, initial vs. combined). The manually corrected segmentation had the highest median number of airways with 179 airways (IQR 167–215) (*p* < 0.001, initial vs. manual) (Fig. 4a). The largest differences were seen in airways from 6th generation onwards (Fig. S2). The tabulated data is presented in Table S2.

### Airway length

Airway length increased with manual correction and retraining. The initial segmentation had a total airway length of 2,319.6 mm (IQR 1905.4–2588.7 mm) which was the lowest amongst all segmentations. This was followed by the combined model segmentation, retrained model segmentation and corrected segmentation, with airway lengths of 2561 mm (IQR 2309.2–3067.3 mm) (*p* = 0.079, initial vs. combined), 2622.2 mm (IQR 2296.1–3492.8 mm) (*p* = 0.051, initial vs. retrained) and 2917.3 mm (IQR 2508.8–3492.8 mm) (*p* < 0.001, initial vs. corrected), respectively (Fig. 4). Airways from the 6th generation onwards showed the largest differences (Fig. S3).

### Airway lumen

Relative to the initial segmentation airway lumen diameters of 5.5 mm (IQR 5.0–5.9 mm), the airway lumen diameters decreased with correction to 5.3 mm (IQR 4.9–5.6 mm) (*p* = 0.009, initial vs. corrected) and the retrained model lumen diameters decreased to 4.9 mm (IQR 4.7–5.5 mm) (*p* = 0.004, initial vs. retrained); however, there was no significant difference between the initial segmentation diameters and the combined model segmentation diameters of 5.0 mm (IQR 4.6–6.1 mm) (*p* = 0.172, initial vs. combined) (Fig. 4c). Detailed breakdown per generation is available in Fig. S4.

## Discussion

We outlined the process for correcting airway segmentations from initial, incomplete segmentations on low-dose CT scans for the purpose of training AI tools. Manual correction resulted into a significantly more complete airway segmentation, and retraining the 3D-Unet resulted into improved segmentations, with the greatest changes seen from the 6th generation onwards. Notably, small airways play an important role in lung diseases such as asthma, COPD, and cystic fibrosis and their accurate detection can be important for the accurate diagnosis and sensitive monitoring of respiratory illness [16, 17]. A focus on improving the segmentation of smaller airways could therefore help in the research of bronchial parameters of early disease [18]. With our methods, it is possible to quickly improve airway segmentations and retrain an AI model.

The research for robust bronchial parameters sometimes includes the evaluation of aggregate measures, such as total airway count and airway tapering [19, 20]. If these measures are obtained from incomplete segmentations, the summary measure may be incorrect. This is illustrated in our study by the decrease in median lumen diameter after correction and retraining. The initial segmentation included too much of the lumen wall and did not include enough of the smaller airways that were visible on the CT scan. This resulted in a significantly larger median airway lumen aggregate measure.

One of the main challenges for AI training in radiology is that often only small, specific datasets from a narrow range of scanning parameters and population characteristics are available for model training and current manual segmentation methods can take up to 15 h to complete for one patient [12]. This makes the design of AI tools that generalise well to data from a broader range of scan parameters and population characteristics very difficult to be built. In turn, pretrained AI models tasked with segmentation may fail when used on data dissimilar to their training dataset. Several AI airway segmentation tools have been reported in the literature, which are typically trained and tested on their own in-house datasets and reference segmentation [21, 22]. However, when deploying the trained AI methods on other data with different characteristics and scanning parameters, their performance may drop drastically [23]. Retraining with use-case specific data allows for the use of AI models in institutions with different scanning techniques.

The aim of DLCST and Erasmus MC-Sophia dataset addition was to improve the AI performance with heterogeneous data, as DLCST scanning protocol differs slightly, and Erasmus MC-Sophia includes paediatric Cystic Fibrosis patients. However, the combined model did not significantly improve AI performance for ImaLife scans.

To segment small airways in low-dose scans or airways beyond occlusions, it typically requires manual intervention. A couple of the scans in our study contained mucous plugging, which prevents segmentation of the airways beyond it when using traditional methods. However, we observed the continuation of segmentation despite large blockages.

A strength of this paper is the use of openly available tools for the methodology. Whilst this technical note focuses on airway segmentations, the same methods can be used to optimise potentially any other segmentation. Our methods are also much less time costly than preparing fully manual airway reference segmentations.

The limitations of this study are the investigation of just one dataset, with a small sample size, based on low-dose CT acquired at high-pitch in a general adult population. Despite the small data-set previous investigation of this, previous investigations of 3D-Unet learning curves shows that models trained with small datasets of just 14 images had just slightly lower performance than the model with 28 images [9]. The manual corrections have been performed by one researcher; within the context of this project, we did not assess the impact of inter-observer variability on the completeness of segmentations.

In conclusion, we showed that openly available software can be used to manually correct initial, incomplete airway segmentations with significant improvement. The resulting segmentations can be used to retrain AI models to increase their efficacy for different scanning protocols and applications. This allows for the quick creation of datasets for AI training that match their use case.

## Supplementary Information

**Additional file 1.** Electronic Supplementary Material Manual

**Additional file 2: Supplementary Table S1.** Brief table of demographic and clinical features

**Additional file 3: Supplementary Table S2.** Tabulated data

**Additional file 4: Supplementary Figure S1.** A flow chart of the trial process

**Additional file 5: Supplementary Figures S2–S4.** Breakdown of results in visual format

## Data Availability

The 3D-Unet used for this research is openly available on github and includes a model file trained on DLCST and Erasmus-MC Sophia datasets. https://github.com/antonioguj/bronchinet

## Abbreviations

3D: Three-dimensional
AI: Artificial intelligence
COPD: Chronic obstructive pulmonary disease
CT: Computed tomography
DLCST: Danish lung cancer screening trial
