# Supplementary figures and images for: Creating a training set for artificial intelligence from initial segmentations of airways
Source: Eur Radiol Exp. 2021 Nov 29;5:54. doi: 10.1186/s41747-021-00247-9 (PMC8627914; doi:10.1186/s41747-021-00247-9)

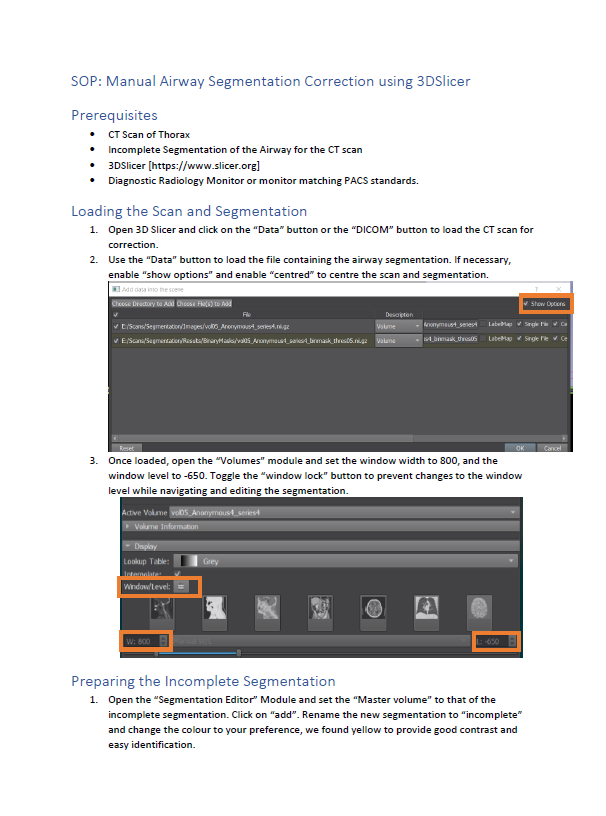

Supplement: Supplementary file 1 — Additional file 1. Electronic Supplementary Material Manual [file 41747_2021_247_MOESM1_ESM.zip › SOP Manual Airway Segmentation.png]

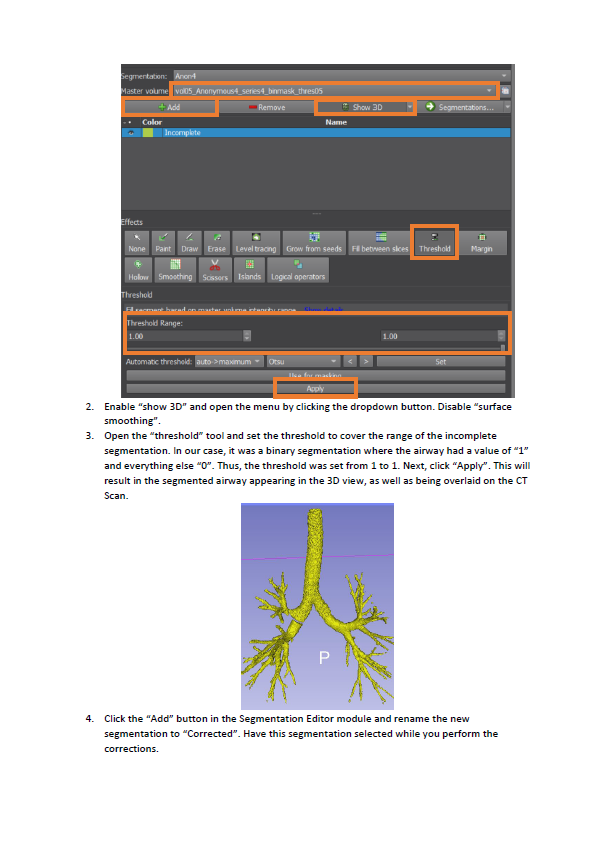

Supplement: Supplementary file 1 — Additional file 1. Electronic Supplementary Material Manual [file 41747_2021_247_MOESM1_ESM.zip › SOP Manual Airway Segmentation1.png]

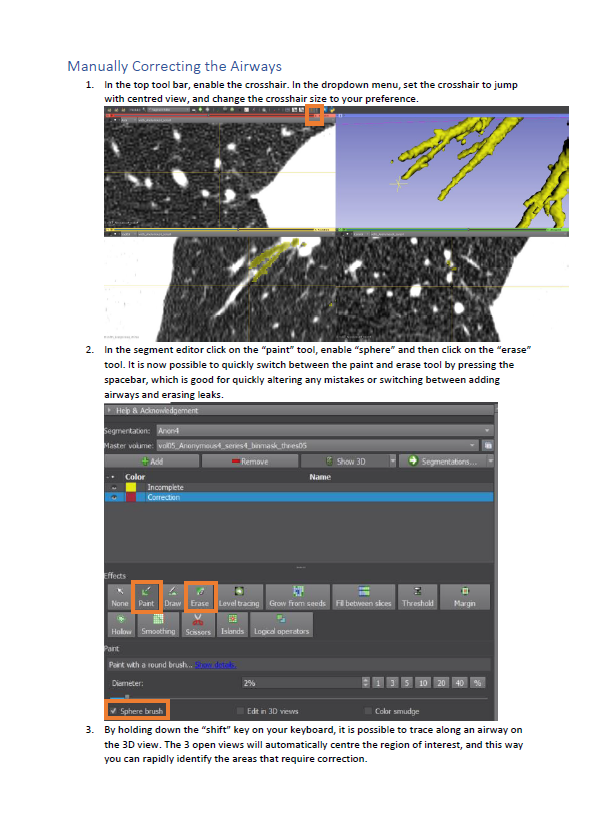

Supplement: Supplementary file 1 — Additional file 1. Electronic Supplementary Material Manual [file 41747_2021_247_MOESM1_ESM.zip › SOP Manual Airway Segmentation2.png]

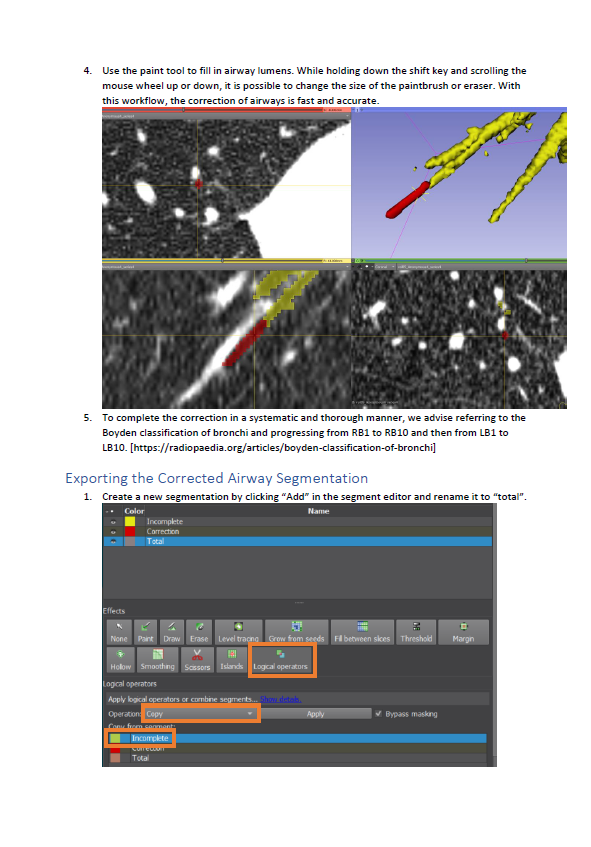

Supplement: Supplementary file 1 — Additional file 1. Electronic Supplementary Material Manual [file 41747_2021_247_MOESM1_ESM.zip › SOP Manual Airway Segmentation3.png]

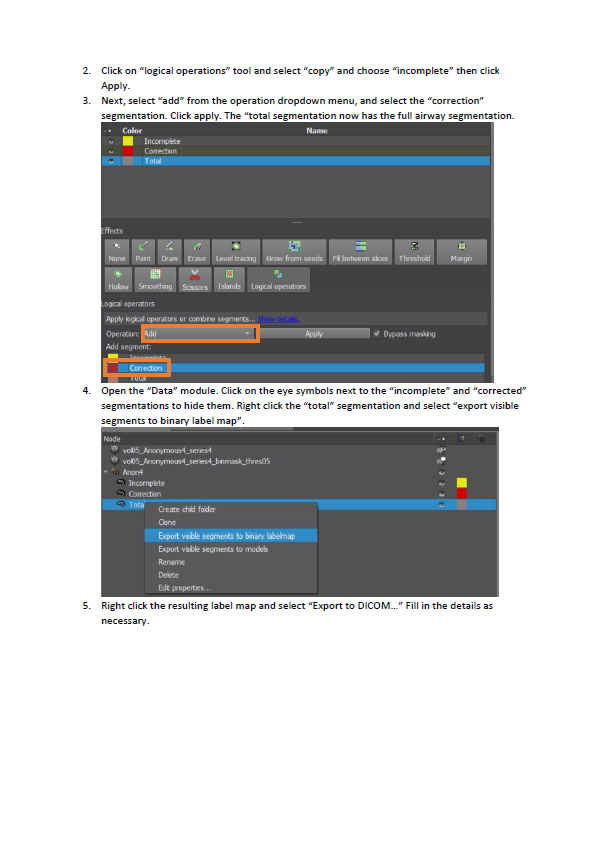

Supplement: Supplementary file 1 — Additional file 1. Electronic Supplementary Material Manual [file 41747_2021_247_MOESM1_ESM.zip › SOP Manual Airway Segmentation4.png]

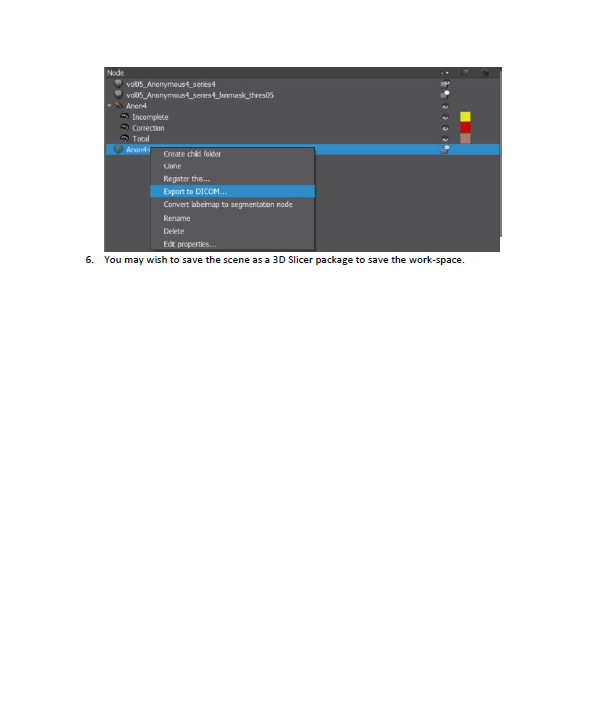

Supplement: Supplementary file 1 — Additional file 1. Electronic Supplementary Material Manual [file 41747_2021_247_MOESM1_ESM.zip › SOP Manual Airway Segmentation5.png]

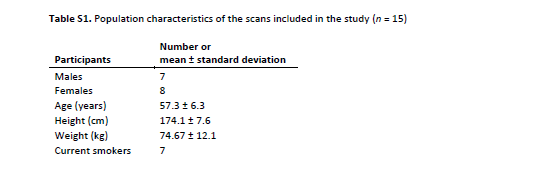

Supplement: Supplementary file 2 — Additional file 2: Supplementary Table S1. Brief table of demographic and clinical features [file 41747_2021_247_MOESM2_ESM.png]

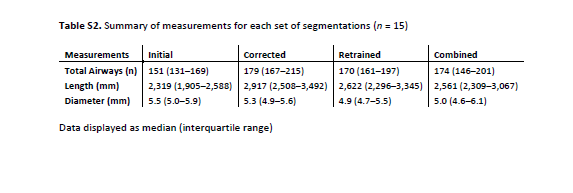

Supplement: Supplementary file 3 — Additional file 3: Supplementary Table S2. Tabulated data [file 41747_2021_247_MOESM3_ESM.png]

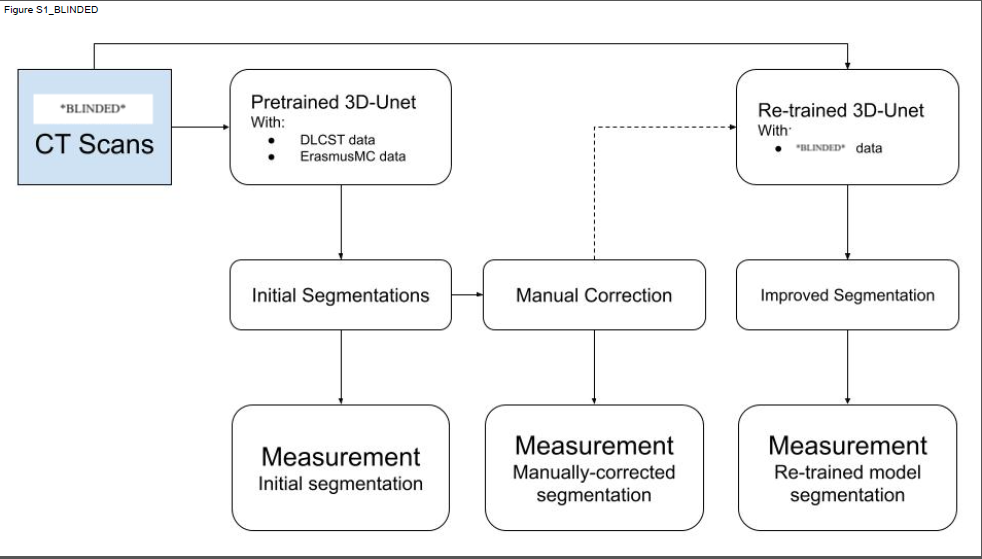

Supplement: Supplementary file 4 — Additional file 4: Supplementary Figure S1. A flow chart of the trial process [file 41747_2021_247_MOESM4_ESM.png]

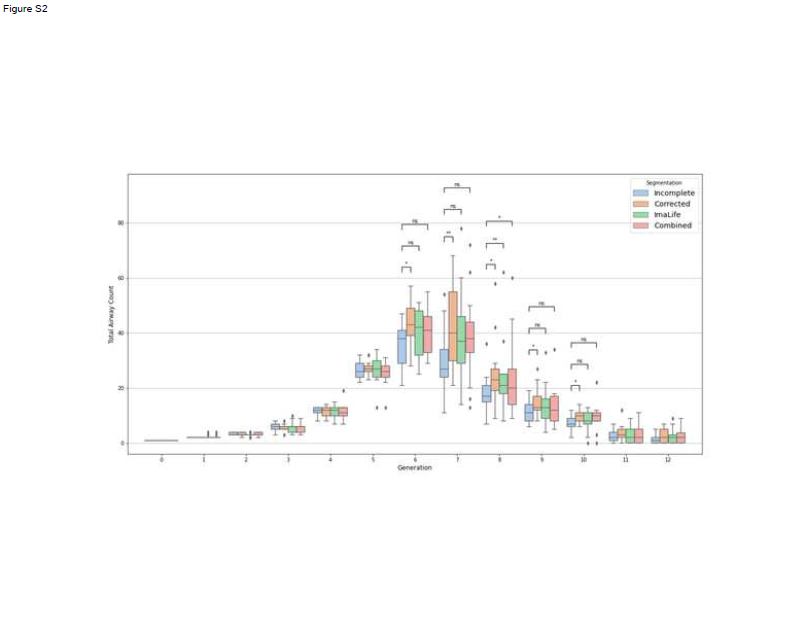

Supplement: Supplementary file 5 — Additional file 5: Supplementary Figures S2–S4. Breakdown of results in visual format [file 41747_2021_247_MOESM5_ESM.zip › Figure S2.png]

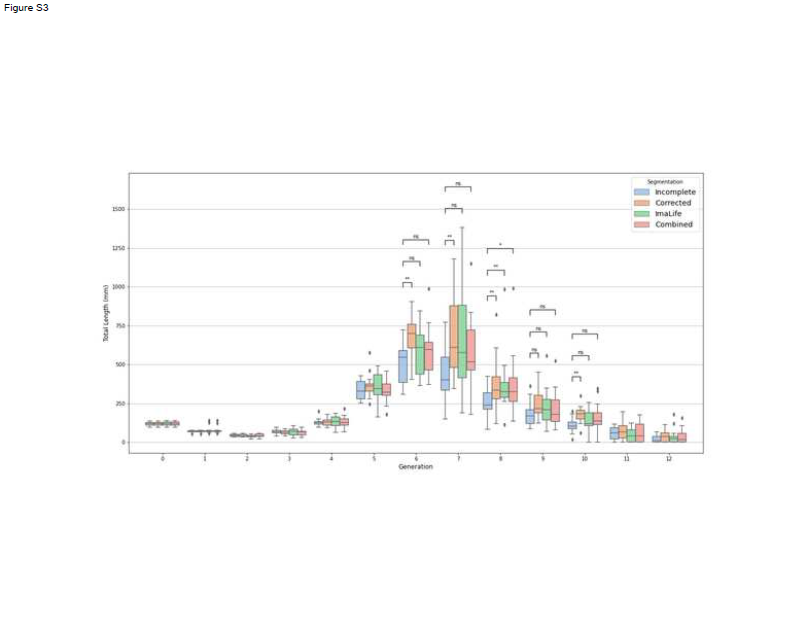

Supplement: Supplementary file 5 — Additional file 5: Supplementary Figures S2–S4. Breakdown of results in visual format [file 41747_2021_247_MOESM5_ESM.zip › Figure S3.png]

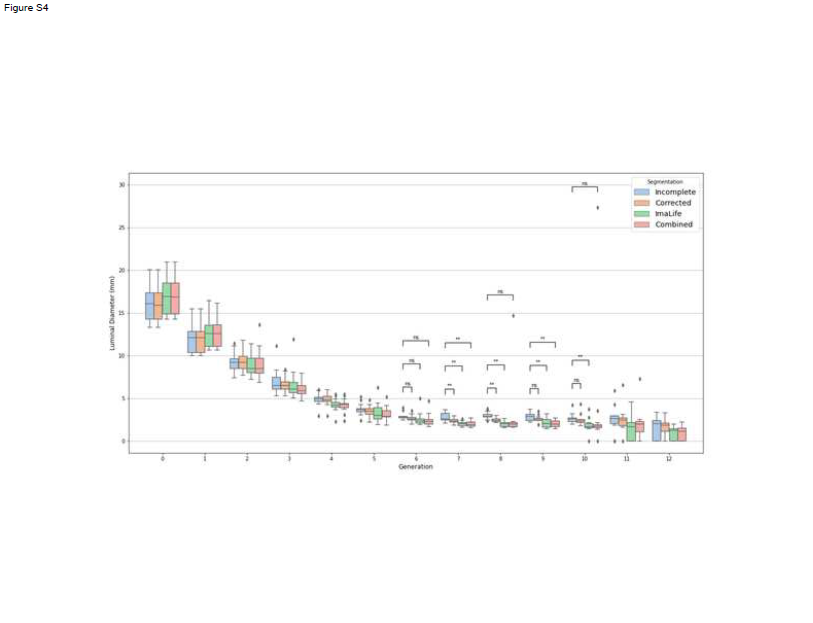

Supplement: Supplementary file 5 — Additional file 5: Supplementary Figures S2–S4. Breakdown of results in visual format [file 41747_2021_247_MOESM5_ESM.zip › Figure S4.png]
